# Case report: Novel use of clinical brain-computer interfaces in recreation programming for an autistic adolescent with co-occurring attention deficit hyperactivity disorder

**DOI:** 10.3389/fnhum.2024.1434792

**Published:** 2024-09-04

**Authors:** Susannah Van Damme, Leslie Mumford, Aleah Johnson, Tom Chau

**Affiliations:** ^1^Clinical Brain Computer Interface Program, Holland Bloorview Kids Rehabilitation Hospital, Toronto, ON, Canada; ^2^Holland Bloorview Kids Rehabilitation Hospital, Bloorview Research Institute, Toronto, ON, Canada; ^3^Institute of Biomedical Engineering, University of Toronto, Toronto, ON, Canada

**Keywords:** brain-computer interface, Autism spectrum disorder, recreation, participation, occupational therapy

## Abstract

**Background:**

In recent years, several autistic^1^ children and youth have shown interest in Holland Bloorview Kids Rehabilitation Hospital’s clinical brain computer interface (BCI) program. Existing literature about BCI use among autistic individuals has focused solely on cognitive skill development and remediation of challenging behaviors. To date, the benefits of recreational BCI programming with autistic children and youth have not been documented.

**Purpose:**

This case report summarizes the experiences of an autistic male adolescent with co-occurring attention deficit hyperactivity disorder using a BCI for recreation and considers possible benefits with this novel user population.

**Methods:**

A single retrospective chart review was completed with parental guardian’s consent.

**Findings:**

The participant demonstrated enjoyment in BCI sessions and requested continued opportunities to engage in BCI programming. This enjoyment correlated with improved Canadian Occupational Performance Measure (COPM) scores in BCI programming, outperforming scores from other recreational programs. Additionally, clinicians observed changes in social communication efforts and self-advocacy in this first autistic participant.

**Conclusion:**

The use of brain computer interfaces in recreational programming provides a novel opportunity for engagement for autistic children and youth that may also support skill development.

## 1 Introduction

Holland Bloorview Kids Rehabilitation Hospital, Canada’s largest children’s rehabilitation hospital, has been implementing brain computer interface (BCI) technology into clinical practice since 2019. The Clinical BCI Program aims to make active play and recreation (e.g., videogames and switch-adapted activities) accessible to all children, agnostic of diagnosis. Initially, most of the children and youth attending the clinic were those with severe neuromotor conditions. However, interest from families of autistic children led to their inclusion in both individual occupational therapy (OT) BCI sessions and therapeutic recreation (TR) BCI group programmes. This case report details the experiences of the first autistic adolescent to participate in both OT and TR BCI sessions, highlighting unexpected benefits observed by clinicians. In healthcare, case reports are retrospective descriptions of single individuals that provide an opportunity to learn from a new phenomenon (Alpi and Evans, 2019). With their focus on the variable interplay between individual, environment and activity, OT and TR are complex interventions well suited to such descriptive reports (McQuaid et al., 2023).

### 1.1 Play

The United Nations Convention on the Rights of the Child (1989) asserts the right of every child to engage in play and recreational activities. In addition to the joy derived from these activities, children and youth develop social, emotional, cognitive, and motor skills by participating in various forms of play (Brown and Lynch, 2023; Rosenberg et al., 2013; Suto, 1998; Tanta and Knox, 2015). Playing as part of a collective encourages greater participation within and beyond the group itself (Gruhl and Lauckner, 2022) and participating in organized activities provides structured opportunities for friendships to develop (Bohnert et al., 2019). For youth with disabilities, group activities enable connection with other people with similar life experiences, supporting their own identity development (Kramer et al., 2015). Despite the importance of play and the moral imperative to provide accessible play opportunities, autistic children and youth continue to have fewer opportunities to participate in recreational activities than their typically developing peers (Hilton et al., 2008; Khalifa et al., 2020; Shannon et al., 2021).

### 1.2 Autism spectrum disorder and attention deficit hyperactivity disorder

Autism spectrum disorder (ASD) is a neurodevelopmental condition characterized by differences in social communication and the presence of restricted, repetitive patterns of behavior, interests, or activities (American Psychiatric Association, 2013). Neurodevelopmental disorders frequently co-occur and specifiers indicating additional clinical characteristics [i.e., associated with attention deficit hyperactivity disorder (ADHD)] are often used within the diagnosis of ASD (American Psychiatric Association, 2013). ADHD is characterized by impairing levels of inattention, disorganization, and/or hyperactivity-impulsivity at levels inconsistent with an individuals’ developmental level (American Psychiatric Association, 2013).

When compared to other children, autistic children and children with ADHD have poorer reported quality of life and mental health (Biggs and Carter, 2016; Clark et al., 2015; Jonsson et al., 2017; Public Health Agency of Canada, 2022). While there are many factors contributing to quality of life and mental health, participation in recreational activity has a well-established positive impact on affect and ability to cope with negative life events (García-Villamisar and Dattilo, 2010; Hutchinson et al., 2008). Additionally, participation in a breadth of organized activities is associated with greater social–emotional adjustment among autistic youth (Bohnert et al., 2019).

Parents of autistic children report that their children have lower rates of participation in home, school, and community environments (Simpson et al., 2018). Social and systemic barriers continue to prevent autistic children from participating in community recreational activities (Gregor et al., 2018). These results point to the need for increased recreational programming tailored to the individual needs and interests of autistic children (Gray, 2017; Gregor et al., 2018).

Caregivers, clinicians, and researchers have identified technology as a motivator for engagement among autistic individuals (Bölte et al., 2010; Frauenberger, 2015; Ghanouni et al., 2020; Scheepmaker et al., 2018). Robot mediated role play, virtual reality and augmented reality systems have been used in interventions to improve narrative skills, reading and social skills (Howorth et al., 2019; Ke et al., 2022; Lorenzo et al., 2019; So et al., 2019). However, few studies have explored the use of technology to facilitate play and recreation. Interactions with technology, be it through a video game, virtual reality console, smart phone app or BCI system, are highly structured and predictable, making them less socially demanding during play. Engagement with technology can therefore be a catalyst for increased human interaction as social pressures are eased and interactions are scaffolded through the rules of technology (Frauenberger, 2015). While clinicians and caregivers posit a danger of dependence on technology (Ghanouni et al., 2020; Frauenberger, 2015) challenges the neurotypical community to explore technology through an autistic lens.

### 1.3 Non-invasive brain computer interfaces

Non-invasive BCIs constitute a novel class of access technologies that are operated independent of physical movement and verbal communication. They enable the control of an external device or application by recording and decoding the user’s brain signals (Wolpaw et al., 2002). BCI applications for adults with physical disabilities include communication, motor restoration/rehabilitation, cognitive rehabilitation, environmental controls, powered mobility, and entertainment (Kinney-Lang et al., 2016; Kirton, 2023; Nicolas-Alonso and Gomez-Gill, 2012; Rashid et al., 2020). While there is less research published on the use of BCIs in pediatrics, children can use them to perform simple tasks (Mikolajewska and Mikolajewski, 2014; Zhang et al., 2019) and may benefit from opportunities that BCIs present to adult populations. BCI research with children with physical disabilities includes exploration of powered mobility (Floreani et al., 2022), augmentative communication (Orlandi et al., 2021), and rehabilitation of hemiparesis (Jadavji et al., 2023). Parents of children with significant physical impairments view non-invasive BCIs as increasing play options and creating potential for collaborative play with peers and siblings (Siu et al., 2024). In contrast, BCI research in autistic populations has focused primarily on neurofeedback training to deliver interventions focusing on symptom reduction through EEG mu rhythm control and cognitive skill development through BCI enabled neurofeedback gaming (Friedrich et al., 2014; LaMarca et al., 2023; Mercado et al., 2019; Pires et al., 2022; Yang et al., 2021). BCI research with children with ADHD focuses on cognitive skill training using video games (Cervantes et al., 2023; Lim et al., 2023). Despite the acknowledged interest in technology within this population, there is no record in the academic literature of BCI-facilitated play or recreation in autistic children with or without ADHD.

## 2 Case description

### 2.1 Clinical brain computer interface program

**Figure 1:**
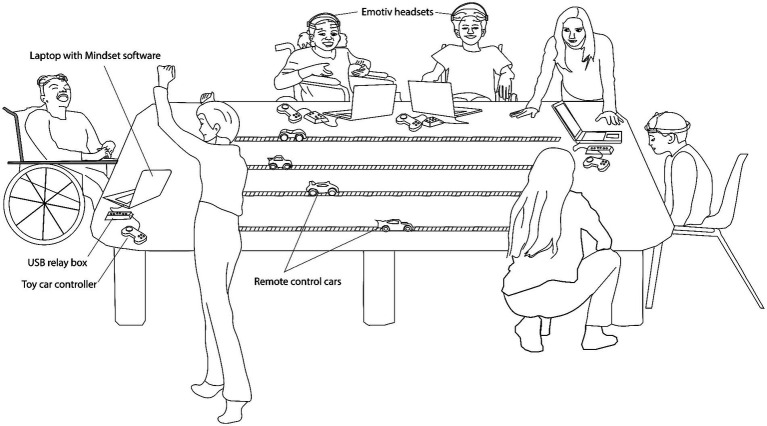
Setup of a typical clinical BCI therapeutic recreation session showing multiple children simultaneously racing remote control toy cars via their mental activity. Each child dons an Emotiv headset. A laptop running the custom Mindset software interfaces with the Emotiv headset to send commands to a custom USB relay box, which in turn controls the toy car remote controller. Staff depicted include two therapeutic recreation specialists and a research assistant.

The clinic uses Emotiv’s Epoc X 14-channel low-cost, wireless, saline headset to detect mental tasks^2^. Electrodes were approximately situated at the following 10–20 locations: AF3, F7, F3, FC5, T7, P7, O1, O2, P8, T8, FC6, F4, F8, AF4. The device samples EEG at 2048 Hz and subsequently down samples the data to 128 Hz. Its bandwidth is 0.16 – 43 Hz. The system does not require gel but uses saline soaked foam pads to interface with the scalp. Participants perform an individualized motor imagery task to activate the BCI, initially receiving the manufacturer-provided visual feedback and subsequently task-specific feedback. Please refer to Taylor and Schmidt (2012) for a detailed discussion of the Emotiv cognitive suite. We invoke a versatile custom-developed, clinician-oriented software application called Mindset (Leung and Chau, 2024) that interfaces with a variety of different EEG headsets, facilitates rapid deployment of many different BCI control paradigms and can send control commands to computer ports or other software applications. Additionally, a custom-built USB relay output box based on a DLP-IOR4 4-channel latching relay output module (DLP Design) connects to a computer via USB port. The Mindset software sends commands via the serial port to activate/deactivate any of the 4 onboard relays. Those relays in turn are connected to switch-accessible devices via 1/8″ mono connectors. Collectively, Mindset and the relay box allow the detections made by the Emotiv software to control switch-accessible apps and toys (See Figure 1). BCI program participants initially work with the clinic’s OT to learn to use the motor-imagery based BCI. Clinicians ask the parent/caregiver about a physical movement that is meaningful to the participant. As such, the actual motor imagery varies among participants. For example, participants may imagine swimming, having a dance party, clapping, jumping, driving their wheelchair, etc. Participants do not interact with a physical object to learn imagery (as in squeezing a ball) but receive copious verbal cueing in the initial sessions. For a neutral state, children are verbally encouraged to have a calm body and mind or quiet thoughts; clinicians speak to the child in a “quiet voice” or sometimes vocalize “shhhh.” Clinician prompting is gradually faded out as the participant learns to independently perform the imagery and neutral tasks over the course of a program. Participants may continue working individually with the OT on BCI-skill related goals, and/or join TR BCI group programming and establish social and recreation-related goals. BCI-specific skills include accurately timing activation to achieve a particular outcome, maintaining a neutral mind to avoid accidental activations of the switch-accessible app or toy, and introducing a “second thought command” to increase possible functions. The latter refers to a second mental activity that the child learns to reliably generate. For example, mental imagery of shooting a basketball can be used to drive a remote-control toy car forward, imagery of clapping for driving in reverse, and calm thoughts to stop the car.

The clinic initially focused on working with children and youth with severe neuromotor conditions. Following a community event in 2022, several autistic children and youth expressed interest in using BCIs and their families expressed enthusiasm for the inclusion of their children in programming. We present here the case study of one such individual who participated in both TR and OT programming. His experience serves as an example of the potential for increased engagement in recreation through use of a BCI for autistic children and youth. With written, informed consent from his parent, we accessed his electronic health record to collect data relating to referral and demographic information, goal setting and participation in BCI and general TR programs.

### 2.2 Participant description

The case participant is a 15-year-old male diagnosed with both autism spectrum disorder, level II (requiring substantial support) and attention deficit hyperactivity disorder. English is his second language, and he communicates verbally using single words or two-to-three-word sentences. His speech is not always intelligible, and he frequently answers questions by repeating the last word spoken. According to clinician observations, the participant frequently reaches out to touch objects within his vicinity. His parent reported that they redirect him by saying “hands quiet” and “hands in pocket.” The participant frequently stands up to wander around during conversations and requires one-to-one support to remain on task. He does not initiate engagement in activity or conversation without staff prompting. He attended programs at a community autism association, but those programs went virtual during the Covid-19 pandemic. He did not engage well with virtual programming.

During the intake process for services at the hospital, the participant’s parent identified goals of learning about options for skill development and encouraging him to be more independent: “The earlier we start will be better for him. I know he struggles with school and he does not enjoy studies. The earlier we can get him into something he enjoys, the better, so that he will be happy.” They described him as very passive and easy-going, rarely initiating an activity or conversation. The participant’s parent also noted that he spends time with his family and does not have friends. They stated “I would like him to practice his listening skills and to respond to questions correctly. I would like him to make friends and be around new people.” When asked directly if he would like to make friends, the participant responded “yes.”

### 2.3 Participant’s engagement with BCI

After trying a BCI at a community event held at the hospital, the participant indicated that he would like to do more BCI activities. Between January 1, 2023, and August 31, 2023, the participant attended a six-week TR BCI group and six individual OT BCI sessions. The participant’s parent reported that they were looking for an activity that he would enjoy and that could build up his confidence. They reported that they were excited about BCIs because the participant had a particular interest in technology. They felt that using a BCI would be motivating for him to engage in meaningful activity.

Table 1 provides a detailed timeline of engagement with BCI programming, goal areas and clinician observations. TR sessions were facilitated by two TR specialists and an assistant on Sunday afternoons with four participants in total. Each week involved a theme with different BCI activities. Please refer to Table 2 for a sample TR session plan and activity list. Following the OT sessions, the family was trained to use the BCI system and borrowed it for home-use. Two virtual BCI OT sessions were scheduled but the participant left each session early.

**Table 1:** BCI programming timeline.

2023	January	February	March	April	May	June	July	August
Programming	TR outpatient group			OT outpatient sessions			Home program with virtual sessions	
Goal areas	Increased control of BCI Making friends			Skill development Increased independence Parent comfort with BCI use			Frequency of BCI use at home	
Clinician observations	5–7 prompts to activate BCI Verbally shared thoughts Made eye contact with peers Moderate staff support to wait his turn Prompts to keep “hands quiet”	3–4 prompts to activate BCI Invited peer to play Frequent smiling Frequent touching of equipment, easily redirected	Independent activation of BCI Eye contact with peers, smiling, laughing, 1–2 word utterances Moderate staff support to wait his turn Enjoys touching objects in room, responds well to redirection	External prompts to activate BCI Looks to parent for reassurance Verbally requests activities	Initiates own verbal prompts to activate BCI Responds to questions appropriately (1–2 word answers) Asks to help Asks for specific activities Parent reported improvements in cognitive and social skills during BCI Sessions	External support for BCI activation Controlled timing of activation for better outcomes Spontaneous conversation unrelated to BCI, 3–4 word sentences Parent set up BCI with support	Participant left during 2 virtual visits Parent report of home use: Working on using BCI more frequently to build confidence and skills By the time headset positioned correctly, no longer wants to wear it Prefers using BCI with others Attempts to remove headset Lower tolerance for sensory input on head when at home	

**Table 2:** Therapeutic recreation activities and session plan.

Component	Description	Duration	BCI enabled activities available TR & OT
Arrival, welcome and introductions	Introductions and question of the day: What is your favorite thing to do using the BCI? Icebreaker game: Would you rather this or that?	15 min	Painting (Sphero robot ball dipped in paint) Music (YouTube or bongo drums) Video games (Alex Runs, Sumo Bootle, Penguin Snowball Fight) Board games (BCI enabled dice roller or BINGO game) Sensory cart BCI-enabled switch toys, bubble machine
Game On!	BCI video games: Alex Runs (activate BCI to make character jump) and Sumo Bootle (time activation of BCI when arrow pointing at target)	10–15 min	
**Time to DJ**	YouTube freeze dance: Activate BCI to turn on music, music plays for 15 s, reactivate to continue playing.	10–15 min	
**Wrap up**	Riddle or Joke (with options) Goodbye(s)	2 min	

BCI sessions started with fitting of the Emotiv Epoc X headset to ensure 100% connectivity with the EEG sensors. The participant then completed 2 cycles of training to establish an active task (or thought command) and a rest task (or neutral mind). Each cycle included two, eight-second recordings of the active task two, eight-second recordings of the rest task. For his active task, the participant chose to imagine jumping and was thus verbally prompted to mentally rehearse this activity. For his rest task, the participant was encouraged to take deep breaths to calm his mind.

Prior to the start of the TR BCI group, TR staff met with the participant and his parent to explore his interests and establish goals for group participation. His parent again reported that the participant would like to make a friend. The participant reported that he would like to make a painting by himself using a BCI. TR staff used a goal menu and the Canadian Occupational Therapy Measure (COPM) to establish and rate individualized goals that relate to group activities (Rezze et al., 2008). The COPM is a well-researched, client-centred outcome measure focusing on self-perceived performance and satisfaction in everyday activities (Carswell et al., 2004). With support from his parent, the participant re-rated his performance and his satisfaction after completion of the group. Table 3 summarizes the participant’s COPM results for the TR BCI group and two additional TR groups he attended, one held prior to BCI programming and one following BCI programming. When comparing pre-and post- group ratings, the participant showed considerably greater improvements in performance and satisfaction with his BCI goal compared to goals in other groups. His performance rating for making a friend improved more in the BCI group while his satisfaction for that goal was consistent with satisfaction in other groups.

**Table 3:** TR group COPM goals and results.

Group details	COPM goal	Importance (baseline)	Baseline	Follow-up	Change
TR Life Skills Hangout 6 sessions, Thursday evenings, Fall 2022	To improve communication skills.	10	Performance: 3	Performance: 5	+2
			Satisfaction: 5	Satisfaction: 7	+2
TR BCI outpatient group 6 sessions, Sunday afternoons, Winter 2023	I will activate the BCI independently	9	Performance: 1	Performance: 10	+9
			Satisfaction: 5	Satisfaction: 10	+5
	I will make a friend	9	Performance: 3	Performance: 9	+6
			Satisfaction: 5	Satisfaction: 7	+2
TR snacks and chat 6 sessions, Tuesday evenings, Fall 2023	I will make a recipe following one step at a time with support.	8	Performance: 4	Performance: 6	+2
			Satisfaction: 5	Satisfaction: 6	+1

The participant initially benefited from verbal cueing to activate the BCI in TR sessions but demonstrated independent activation by the end of the group. In subsequent OT sessions, he again required verbal cueing to activate the BCI. Across both TR and OT sessions, the participant showed improvement in his ability to control the timing of his activation and to maintain a neutral state. These changes are reflected in both COPM scores and clinical observations. In his fifth onsite OT session, the participant used the BCI to release a numbered ball from a Bingo machine. He then maintained a neutral state to pick up the ball, read out the number and place a chip on his Bingo card. After indicating that he wanted the game to move more quickly, the participant intentionally activated the Bingo machine to release three balls in succession, demonstrating increased control of the BCI and of game play itself.

While clinicians did not note changes in impulsivity (e.g., continued reaching out to touch materials) or attention (as seen by ongoing need for single step instruction), they did observe increases in spontaneous social communication, self-advocacy, and independent engagement. Throughout TR sessions, the participant made eye contact and smiled at peers. In the fourth TR session, he spontaneously asked a peer to play catch with him in a “friendly way.” Throughout OT sessions, he answered questions directly, made requests for activities, indicated when he was finished playing and offered to help set-up and clean-up activities. In his final onsite OT session, the participant “actively engaged in conversation with the Clinical BCI team. [He] asked questions (i.e., ‘Can I go now?’), advocated for himself (i.e., ‘I want/ do not want this’), and shared events from his life (i.e., ‘My uncle is coming!’)”

The home loan of BCI equipment was initiated to encourage recreation in a non-clinical environment. The OT recommended a family game night to focus on fun and socialization. The participant’s parent expressed wanting him to continue to use a BCI to develop cognitive skills and set a goal for using it three times per week. However, in follow-up sessions with the OT, the parent reported that the participant lacked motivation to use the BCI due to sensitivity to touch on his head and changes in routine. The participant became increasingly intolerant of using the BCI at home and it was returned after 2 months. When asked by his parent whether he would like to continue using a BCI, the participant indicated yes, but not at home.

The participant continues to engage in BCI programming, both as part of TR groups and individual OT sessions. He is now gaining experience with more independent participation by being dropped off for his sessions by his parents.

## 3 Discussion

Brain computer interfaces offer the opportunity to engage with the world without requiring speech or physical movement. As such, they are expected to increase play opportunities for children with severe physical disabilities (Siu et al., 2024). Autistic children and youth are not necessarily reliant on technology to participate in recreation but there may be unexpected benefits of technology-mediated recreational programming for this population.

Many people enjoy the novel cognitive challenge and futuristic appeal of BCIs. However, this initial interest wanes quickly for individuals who have other means of engaging in recreation. We expected that the same would be true for our participant given his ample physical abilities. Instead, he expressed clear interest in ongoing participation in BCI programming and demonstrated improvements in both BCI skill and pro-social behaviors. The return to needing support for activation during OT sessions may indicate decreased skill retention following the end of the TR group. However, skill generalization from one environment to another is a known challenge for autistic individuals (Brown and Bebko, 2012). The participant may have had difficulty initiating BCI activation outside of the structure of the TR program.

Increased COPM satisfaction scores suggest that improvements were meaningful to the participant. The perfect satisfaction score for BCI skill aligns with his ability to speed up game play when he wanted to finish a game quickly. Similarly, his increased scores for his goal to make a friend may be reflective of increased ease with social communication observed throughout the TR group. These results suggest that there is value to the inclusion of BCI technology in recreational programming for autistic children and youth even if they have at their disposal, more direct means of accessing recreational activities.

Frauenberger’s work provides some insight into why the participant experienced unexpected gains. In 2015, he asked “Has our focus on delivering interventions obstructed our view on what could be the real power of interactive technology in the lives of autistic people?” In co-designing technologies with autistic children, Makhaeva et al. (2016) developed the concept of *Handlungsspielraum*: the space for creative action and exploration created from the tension between structure and creative freedom. The BCI set-up and recreational programming were highly structured and predictable. More importantly, the BCI-related circumvention of the motor system likely suppressed the reafferent sensory signal associated with voluntary movement (Brincker and Torres, 2018), thereby reducing the uncertainty of feedback to the central nervous system (Brincker and Torres, 2013). Collectively, these constraints may have afforded the scaffolding necessary for our participant to engage in spontaneous conversation, advocate for his preferred activities, offer to help clean up, and have fun.

When the original structure of the participant’s engagement with a BCI shifted to the home environment, his interest in using a BCI decreased significantly. Several factors may account for this change. Virtual OT sessions were likely a poor fit given the participant’s response to virtual programming elsewhere. The participant may have had a different sensory experience with the headset at home or a difference in motivation to use the headset, impacting his ability to tolerate it. The shift in goals from a focus on BCI mastery and social participation during clinical sessions to frequency of use at home may have also impacted enjoyment of BCI activities. Finally, the lack of peer interactions and opportunities for group engagement may have made BCI use at home less enjoyable.

The reluctance to use a BCI at home suggests that the technology itself was not the prime motivator for engagement. However, his COPM scores indicate that engagement with the TR BCI outpatient group was more impactful than other TR-based groups attended. That the participant chose to continue engaging in BCI activities by signing up for a subsequent TR BCI outpatient group and individual OT sessions supports the value of this type of programming outside of the home. His interests in external programming are consistent with the research literature indicating the benefits of engagement in organized activities (Gruhl and Lauckner, 2022; Bohnert et al., 2019).

While we cannot conclude that the observed changes in the participant’s engagement were exclusively due to BCI use, the participant’s COPM results indicate that he benefited more from the BCI recreation program than from the highly structured Life Skills Hangout and Snacks and Chat programs. This case study illustrates that a BCI program can afford sorely needed opportunities for participation in recreation for this population. As such, our clinical BCI program will continue to welcome autistic children and youth.

The participant’s family only consented to a retrospective examination of the participant’s recreation and BCI-related health record. As such, we did not have access to the participant’s full medical record and cannot report on physical examination results or more detailed diagnostic information.

Future research could compare outcomes prospectively between traditional and BCI recreation programs and between BCI and other technology-focused offerings, such as a robotics program.

## 4 Conclusion

Our case study participant exhibited higher enjoyment scores in BCI programming than in his previous recreational programs, and clinicians observed improvements in social communication and self-advocacy. It is the nature of case reports that any conclusions may only apply to the case subject being reported. As clearly stated by autism advocate and professor of special education, Dr. Stephen Shore: “If you have met one person with autism, you have met one person with autism” (Flannery and Wisner-Carlson, 2020). So, while we cannot generalize our observations, we can contribute to the growing literature supporting the recreational use of non-invasive BCIs in pediatrics and encourage clinicians to offer this type of programming to autistic children and youth. Using a participatory research lens and including autistic children and youth, and their families in the planning and execution of future recreational BCI research will ensure relevance, value, and effective implementation (Fletcher-Watson et al., 2019).

## Data Availability

The original contributions presented in the study are included in the article/supplementary material, further inquiries can be directed to the corresponding authors.

## Footnotes

^2^ https://www.emotiv.com/products/epoc-x
